# Radial Nerve Palsy Associated with Humeral Shaft Fractures: Incidence, Recovery Patterns, and Functional Outcomes in Surgically Treated Patients

**DOI:** 10.3390/medicina62071427

**Published:** 2026-07-22

**Authors:** Ahmet Acar, Ahmet Berkay Girgin, Ayşe Betül Acar, Muhammed Fazıl Özcan, Ömer Torun

**Affiliations:** 1 Department of Orthopedics and Traumatology, Etlik City Hospital, 06170 Ankara, Turkey; fazilozcan00@gmail.com (M.F.Ö.); omertor46@gmail.com (Ö.T.); 2 Department of Orthopedics and Traumatology, Akyurt State Hospital, 06750 Ankara, Turkey; abgirgin1995@gmail.com; 3 Department of Algology and Pain Medicine, Etlik City Hospital, 06170 Ankara, Turkey; betulbozann@gmail.com

**Keywords:** humeral shaft fracture, radial nerve palsy, radial nerve recovery, postoperative radial nerve palsy, QuickDASH, nerve exploration

## Abstract

*Background and Objectives*: Radial nerve palsy is one of the most clinically important neurological complications associated with humeral shaft fractures. This study aimed to evaluate the incidence of radial nerve palsy in adult humeral shaft fractures and to investigate recovery patterns and functional outcomes in surgically treated patients. *Materials and Methods*: This single-center retrospective observational study included adult patients with humeral shaft fractures treated between November 2022 and January 2025. All adult humeral shaft fractures during the study period were screened to determine the overall rate of radial nerve palsy. The final functional analysis included surgically treated patients with radial nerve palsy. Demographic data, fracture characteristics, injury energy, fracture type, timing of radial nerve palsy, treatment modality, operative time, radial nerve exploration, additional surgery, time to nerve recovery, and QuickDASH scores were evaluated. Conservatively managed patients with radial nerve palsy were included in the calculation of overall incidence and recovery rates but were not included in the final functional analyses. *Results*: A total of 469 adult patients with humeral shaft fractures were evaluated. Radial nerve palsy was identified in 77 patients, corresponding to an overall rate of 16.4%. The rate was 46.4% in open fractures and 14.5% in closed fractures. Among the 77 patients with radial nerve palsy, 49 underwent surgical treatment and constituted the final analysis cohort, whereas 28 were managed conservatively. Recovery was observed in 25 of 28 conservatively managed patients (89.3%) and in 35 of 49 surgically treated patients (71.4%). Overall, radial nerve function recovered in 60 of 77 patients (77.9%). New-onset postoperative radial nerve palsy developed in 29 of 129 surgically treated patients without preoperative palsy (22.5%); however, recovery occurred in 22 of these 29 patients (75.9%), and the rate of persistent postoperative palsy was 5.4% among patients without preoperative palsy. In the surgical cohort, open fracture, longer operative time, and higher initial Cobb angle were associated with non-recovery. Recovery of radial nerve function was the only factor independently associated with better QuickDASH scores. *Conclusions*: Radial nerve palsy was more frequent in open humeral shaft fractures. Although recovery was high among conservatively managed patients, surgically treated cases showed a lower recovery rate, likely reflecting greater injury complexity. New-onset postoperative radial nerve palsy was relatively frequent, but most cases recovered. Recovery of radial nerve function was strongly associated with better upper extremity functional outcomes.

## 1. Introduction

Humeral shaft fractures have an important place among long-bone fractures observed in the adult population and account for approximately 1–5% of all humeral fractures [1]. The diaphyseal region of the humerus is an anatomical area that should be carefully evaluated not only in terms of bone healing and functional outcomes but also because of the risk of involvement of adjacent neurovascular structures. The radial nerve is the most clinically important neurological structure in this region. Because the radial nerve courses close to the bone along the spiral groove on the posterior aspect of the humerus and passes through the lateral intermuscular septum in the distal third of the arm to enter the anterior compartment, it is particularly vulnerable to injury in humeral shaft fractures [2]. The reported incidence of radial nerve palsy associated with humeral shaft fractures is approximately 7–17% in the literature [3].

Radial nerve palsy may occur as a result of direct nerve injury at the time of trauma, displacement of fracture fragments, traction, or entrapment of the nerve at the fracture site [4]. In addition, iatrogenic radial nerve injury may occur during surgical treatment [5,6]. Therefore, evaluation of radial nerve function is an important component of clinical follow-up and treatment planning both at initial presentation and in the postoperative period. Although a substantial proportion of radial nerve palsies are neuropraxic in nature and may recover spontaneously over time, the presence of nerve injury can directly affect the surgical approach, the decision for exploration, the rehabilitation process, and the patient’s functional outcome [7,8].

In addition to conservative methods, surgical treatment options such as plate osteosynthesis and intramedullary nailing are widely used in the management of humeral shaft fractures [9]. However, there is still no clear consensus in the literature regarding routine exploration of the radial nerve in surgically treated patients [10]. Some authors advocate early exploration in the presence of radial nerve palsy to assess nerve continuity and identify possible nerve entrapment or laceration, whereas other studies report that clinical and electrophysiological follow-up may be sufficient for many patients, particularly in closed fractures [4,11,12]. This uncertainty makes the treatment approach and follow-up strategy for patients with humeral shaft fractures and radial nerve palsy an important area of clinical debate.

In patients with radial nerve palsy, neurological recovery is thought to be related not only to the type of nerve injury but also to fracture characteristics and the surgical method used. Fracture pattern, injury energy, the presence of an open fracture, the distance of the fracture from the proximal and distal joint, angulation between fracture fragments, whether radial nerve exploration is performed, and electrophysiological findings are among the factors that may influence the recovery process [13]. Nevertheless, studies evaluating the rate and time of nerve function recovery, need for additional surgery, electromyography findings, and functional outcomes together in patients with radial nerve palsy remain limited.

The primary aim of this study was to evaluate the recovery rate and time to recovery of radial nerve function in patients who underwent surgical treatment for humeral shaft fractures and developed radial nerve palsy. The secondary aim was to investigate the association of factors such as injury energy, fracture type, fracture location, angulation between fracture fragments, surgical method, radial nerve exploration, electromyography findings, and the need for additional surgery with neurological recovery and functional outcomes.

## 2. Materials and Methods

### 2.1. Study Design and Patient Selection

This study was designed as a single-center, retrospective, observational study. Adult patients who underwent surgical treatment for humeral shaft fractures and were diagnosed with radial nerve palsy during the follow-up period between 1 November 2022 and 1 January 2025 were included.

Patients older than 18 years who underwent surgical treatment for a humeral shaft fracture, had radial nerve palsy, and had accessible clinical and radiological follow-up data were included in the study. Patients with fractures outside the humeral shaft, intra-articular proximal or distal humeral fractures, those younger than 18 years of age, and those with missing clinical, radiological, electrophysiological, or functional data required for the study were excluded.

During the same study period, all adult patients with humeral shaft fractures were also screened to determine the overall rate of radial nerve palsy. However, the final outcome analysis included only patients with radial nerve palsy who underwent surgical treatment. Patients with radial nerve palsy who were managed conservatively were used for calculating the overall rate of radial nerve palsy and the overall recovery rate; however, they were not included in the final functional analyses or in the analyses of surgical variables.

Ethical approval for this study was obtained from the Ankara Etlik City Hospital Clinical Research Ethics Committee (Approval no: 2026-188, dated 18 February 2026). The study was conducted in accordance with the principles of the Declaration of Helsinki. Because of the retrospective design of the study, the requirement for informed consent was waived.

### 2.2. Data Collection

Patient data were obtained retrospectively by reviewing the hospital information system, patient files, operative notes, and available radiological images. Age, sex, and fracture side were recorded as demographic data. Fracture and trauma characteristics included whether the fracture was open or closed, injury energy, fracture pattern, and anatomical location of the fracture. Fracture pattern was classified as transverse, spiral/oblique, or comminuted. Injury energy was recorded as low-energy or high-energy trauma.

The fixation method, operative time, whether radial nerve exploration was performed, and the need for additional surgery after radial nerve palsy were recorded as variables related to the surgical process. Fixation method was classified as plate osteosynthesis or intramedullary nailing. Intraoperative findings were evaluated from operative notes in patients who underwent radial nerve exploration. Additional surgical procedures performed after radial nerve palsy, such as secondary exploration, nerve repair, nerve grafting, tendon transfer, or bone osteotomy, were also recorded.

**Figure 1:**
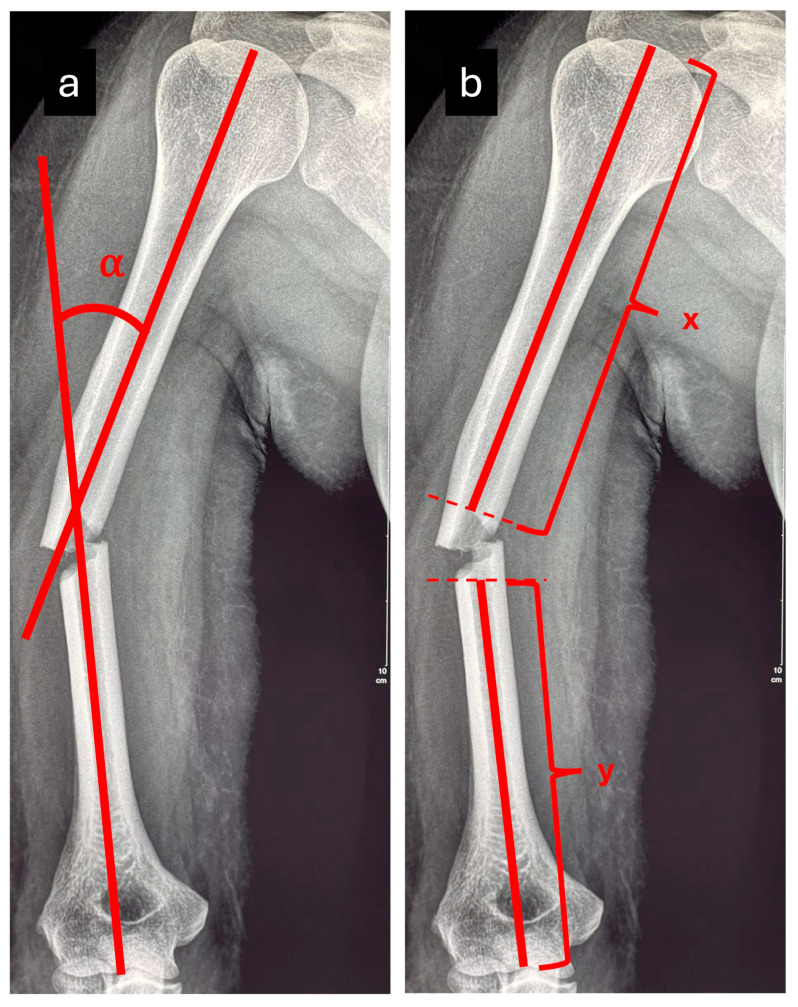
(**a**) The Cobb angle (α) was measured as the angle between the longitudinal axes of the proximal and distal fracture fragments on the anteroposterior radiograph. (**b**) The proximal fracture distance (x) was measured from the proximal humeral articular surface to the most proximal extent of the fracture, whereas the distal fracture distance (y) was measured from the distal humeral articular surface to the most distal extent of the fracture.

Radiological evaluation was performed using the available radiographs obtained at presentation and during follow-up. The distances of the fracture from the proximal and distal joints were measured in millimeters. The proximal fracture distance (X) was defined as the distance between the proximal humeral articular surface and the most proximal extent of the fracture, whereas the distal fracture distance (Y) was defined as the distance between the distal humeral articular surface and the most distal extent of the fracture. In addition, the angulation between the proximal and distal fracture fragments on the initial radiographs was measured using the Cobb angle method. This measurement was used to assess whether angulation between the fracture fragments might create a traction effect on the radial nerve (Figure 1).

Radial nerve palsy was defined as the presence of motor and/or sensory loss in radial nerve function on clinical examination. Whether radial nerve palsy was present at initial presentation after trauma or developed in the postoperative period was recorded. In patients who underwent electromyography, EMG findings were reviewed, and the level and severity of nerve injury were recorded when available.

Whether radial nerve function recovered and the time to recovery were evaluated from patient files and follow-up records. Time to recovery of nerve function was defined as the time at which clinically meaningful improvement in radial nerve motor function was first documented. Clinically meaningful improvement was defined as the first documented recovery of active wrist and finger extension against gravity (Medical Research Council grade ≥ 3) together with progressive improvement in sensory function within the radial nerve distribution. Splint use, duration of physical therapy and rehabilitation, need for additional treatment, and functional outcomes were also recorded. Functional status was evaluated using the Quick Disabilities of the Arm, Shoulder and Hand (QuickDASH) score. The QuickDASH is an 11-item patient-reported outcome measure assessing upper extremity disability. Scores range from 0 to 100, with lower scores indicating better function and higher scores indicating greater disability.

### 2.3. Statistical Analysis

Statistical analyses were performed using IBM SPSS Statistics for Windows, Version 27.0 (IBM Corp., Armonk, NY, USA). The normality of continuous variables was assessed using the Shapiro–Wilk test. Continuous variables that did not follow a normal distribution were presented as the median and interquartile range (IQR), whereas categorical variables were expressed as frequencies and percentages. Comparisons of continuous variables between two independent groups were performed using the Mann–Whitney U test. Associations between categorical variables were evaluated using Pearson’s chi-square test or Fisher’s exact test, as appropriate. Factors associated with recovery of radial nerve function were analyzed using univariate comparisons between patients with and without nerve recovery. To identify independent predictors of functional outcome, a multivariable linear regression analysis was performed with the Quick Disabilities of the Arm, Shoulder and Hand (QuickDASH) score as the dependent variable. Variables considered clinically relevant or showing significance in univariate analyses were included in the regression model. Regression coefficients (B), standardized beta coefficients (beta), 95% confidence intervals (CI), and *p* values were reported. A two-tailed *p* value < 0.05 was considered statistically significant.

## 3. Results

**Figure 2:**
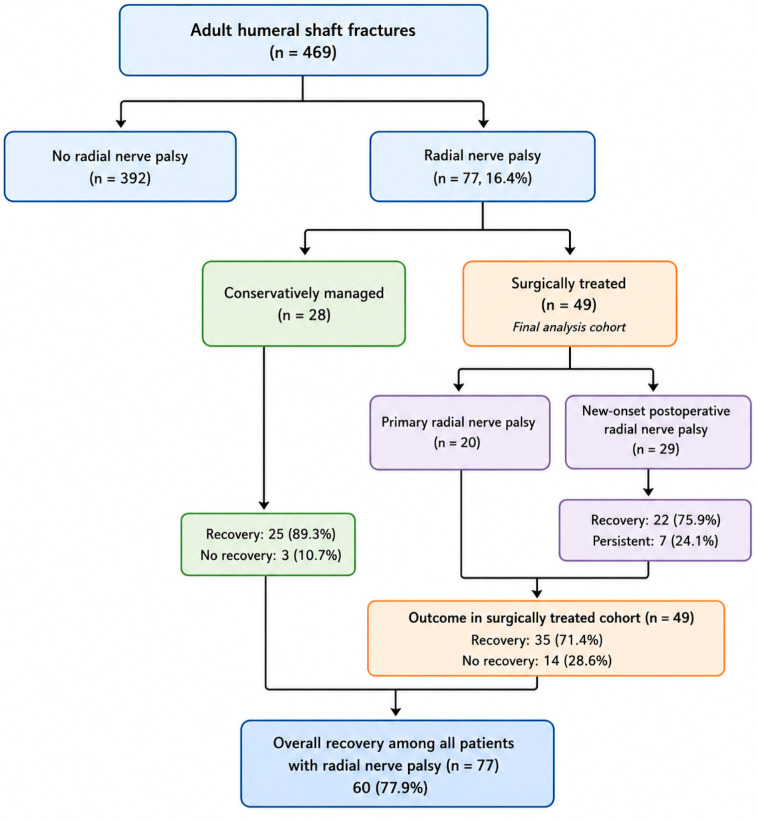
Study flow chart.

During the study period, a total of 469 adult patients with humeral shaft fractures were evaluated. Of these, 28 had open fractures and 441 had closed fractures. Radial nerve palsy was identified in 77 patients, corresponding to an overall radial nerve palsy rate of 16.4%. Radial nerve palsy was observed in 13 of 28 patients with open humeral shaft fractures and in 64 of 441 patients with closed humeral shaft fractures. Among the 77 patients with radial nerve palsy, 49 underwent surgical treatment and constituted the final analysis cohort of the present study, whereas 28 patients were managed conservatively and were excluded from the final functional analyses and analyses of surgical variables. Recovery of radial nerve function was observed in 25 of the 28 conservatively managed patients (89.3%). When surgically and conservatively managed patients were considered together, radial nerve function recovered in 60 of 77 patients, corresponding to an overall recovery rate of 77.9% (Figure 2).

The final analysis cohort consisted of 49 surgically treated patients with radial nerve palsy associated with humeral shaft fractures. The median age of the patients was 33 years (IQR: 22.5–50.5), and 33 patients (67.3%) were male. High-energy trauma was present in 31 patients (63.3%), whereas low-energy trauma was present in 18 patients (36.7%). Open fractures were identified in 13 patients (26.5%), and closed fractures in 36 patients (73.5%).

Radial nerve palsy occurred after trauma in 20 patients (40.8%) and in the postoperative period in 29 patients (59.2%). The most commonly used treatment method was plate fixation, which was performed in 47 patients (95.9%); intramedullary nailing was performed in 2 patients (4.1%). In terms of fracture pattern, 14 fractures (28.6%) were transverse, 17 (34.7%) were spiral/oblique, and 18 (36.7%) were comminuted. The median Cobb angle was 24 degrees (IQR: 16–33.5), and the median operative time was 100 min (IQR: 80–120).

Among the 29 patients who developed new-onset postoperative radial nerve palsy, recovery of radial nerve function was observed in 22 patients (75.9%), whereas no recovery was observed in 7 patients (24.1%). Among the 22 patients who recovered, the mean time to recovery was 121.2 days (range: 30–500 days). Accordingly, the rate of persistent postoperative radial nerve palsy was 5.4% among surgically treated patients without preoperative radial nerve palsy (7 of 129 patients).

Radial nerve exploration was performed in 34 patients (69.4%), and 24 patients (49.0%) required additional surgery. Among the 24 patients who required additional surgery, some underwent more than one procedure at different time points. Additional procedures included nerve grafting in 6 cases, tendon transfer in 5, surgery for nonunion in 6, plate fixation after external fixation in 8, revision open reduction and internal fixation in 3, and additional fixation for non-humeral fractures in 5. Clinical recovery of radial nerve function was observed in 35 patients (71.4%), whereas no recovery was observed in 14 patients (28.6%). Among patients with recovery, the mean time to recovery was 136.9 days (range: 21–500 days), with a standard deviation of 117.6 days. The median QuickDASH score was 37.0 (IQR: 22.9–55.5) (Table 1).

QuickDASH scores were compared according to clinical characteristics. No significant differences in QuickDASH scores were found according to sex, injury energy, fracture type, timing of radial nerve palsy, nerve exploration, or the presence of additional surgery. In contrast, the mean rank of the QuickDASH score was significantly higher in patients without recovery of radial nerve function than in those with recovery. This finding indicated worse functional outcomes in the non-recovery group (34.36 vs. 21.26, *p* = 0.004) (Table 2).

To evaluate factors associated with recovery of radial nerve function, patients with and without neurological recovery were compared. Radial nerve function recovered in 35 patients, whereas no recovery was observed in 14 patients. Sex, injury energy, timing of radial nerve palsy, treatment modality, fracture pattern, nerve exploration, presence of additional surgery, age, distal fracture distance, and proximal fracture distance were not significantly associated with recovery of radial nerve function.

The rate of open fractures was significantly higher in the non-recovery group than in the recovery group (50.0% vs. 17.1%, *p* = 0.031). The median operative time was also significantly longer in patients without nerve recovery than in those with recovery (120 min [IQR: 100–140] vs. 90 min [IQR: 75–110], *p* = 0.033). In addition, the median Cobb angle was significantly higher in patients without recovery of nerve function than in those with recovery (33.5 degrees [IQR: 21–45] vs. 23 degrees [IQR: 15–30], *p* = 0.026). Functional outcomes were also significantly worse in patients without nerve recovery. The median QuickDASH score was 72.7 (IQR: 45.5–79.5) in the non-recovery group and 34.0 (IQR: 22.4–46.5) in the recovery group (*p* = 0.004) (Table 3).

A multivariable linear regression analysis was performed to identify factors associated with the QuickDASH score. Age, operative time, Cobb angle, presence of an open fracture, and recovery of radial nerve function were included in the model. Among these variables, recovery of radial nerve function was the only significant determinant of the QuickDASH score. Recovery of radial nerve function was associated with a 23.027-point decrease in the QuickDASH score, indicating a better functional outcome (B: −23.027, 95% CI: −38.314 to −7.740, standardized beta: −0.467, *p* = 0.004). Although age showed a borderline association with the QuickDASH score, it did not reach statistical significance (B: 0.303, 95% CI: −0.027 to 0.633, *p* = 0.071). Operative time, Cobb angle, and the presence of an open fracture were not independently associated with the QuickDASH score (Table 4).

## 4. Discussion

The main findings of this study indicate that radial nerve palsy is a clinically important problem in patients with humeral shaft fractures and that recovery of radial nerve function has a substantial impact on functional outcomes. Among 469 adult patients with humeral shaft fractures evaluated during the study period, the overall rate of radial nerve palsy was 16.4%. This rate was 46.4% in open fractures and 14.5% in closed fractures. Of the 77 patients with radial nerve palsy, 49 underwent surgical treatment and constituted the final functional analysis cohort. Among the 28 patients who were managed conservatively, radial nerve function recovered in 25 patients, corresponding to a recovery rate of 89.3% in this group. In contrast, the recovery rate was 71.4% in the surgically treated group. When all patients with radial nerve palsy were evaluated together, the overall recovery rate was 77.9%. In the surgical cohort, the non-recovery group had a higher rate of open fractures, longer operative time, and a higher initial Cobb angle. In addition, recovery of radial nerve function was associated with better QuickDASH scores.

In the literature, the reported rate of radial nerve palsy associated with humeral shaft fractures generally ranges between 7% and 17% [3,14,15]. In the systematic review by Hegeman et al., the incidence of primary radial nerve palsy among 4972 humeral shaft fractures was reported as 12.2% [14]. In the systematic review by Hendrickx et al., which evaluated closed humeral shaft fractures, the rate of primary radial nerve palsy was 10% [15]. Schwab et al. reported an overall radial nerve palsy rate of 19% among patients with humeral shaft fractures treated with internal fixation [3]. The overall radial nerve palsy rate of 16.4% in our study is therefore consistent with the upper range of previously reported rates. This relatively high rate may be related to the fact that the study was conducted in a tertiary trauma center, the relatively high proportion of open fractures and high-energy trauma, and the important contribution of surgically treated complex cases to the study population.

One notable finding of our study was that the rate of radial nerve palsy was markedly higher in open fractures than in closed fractures. The rate of radial nerve palsy was 46.4% in open fractures, compared with 14.5% in closed fractures. This finding may be explained by the fact that open fractures are usually associated with higher-energy trauma, greater soft tissue damage, more pronounced fracture displacement, and a higher likelihood of direct nerve injury. Ring et al., in their study evaluating radial nerve palsy associated with high-energy humeral shaft fractures, reported that nerve transections were particularly associated with complex open injuries [16]. Similarly, the literature suggests that open fractures, penetrating injuries, vascular injuries, or severe soft tissue damage may be associated with more serious radial nerve injury and may more strongly support early exploration [16,17]. In our study, the higher rate of open fractures in the non-recovery group also suggests that open fracture may represent an unfavorable clinical marker not only for the development of radial nerve palsy but also for nerve recovery.

In the present study, the recovery rate among conservatively managed patients with radial nerve palsy was 89.3%. This rate is consistent with the high spontaneous recovery rates reported in the literature for primary radial nerve palsy associated with closed humeral shaft fractures. Bumbasirevic et al., in their series of 117 patients with humeral shaft fractures and radial nerve palsy, reported spontaneous nerve recovery rates of 95% in closed fractures and 94% in low-grade open fractures [18]. Similarly, Hendrickx et al. reported that more than 90% of primary radial nerve palsies associated with closed humeral shaft fractures recovered without the need for reintervention [15]. Therefore, the recovery rate observed in our conservatively managed group supports the continued relevance of initial observation and clinical follow-up in closed fractures and appropriately selected patients.

By contrast, the recovery rate of radial nerve function in the surgically treated final analysis cohort was 71.4%. This rate was lower than that observed in the conservatively managed group and in some previously published series. Hegeman et al. reported an overall radial nerve palsy recovery rate of 85.8% in their systematic review [14]. In the prospective HUMMER study by Van Bergen et al., recovery rates were reported as 94% for radial nerve palsy present at the time of trauma and 89% for postoperative radial nerve palsy [19]. In our study, when all patients with radial nerve palsy were considered together, the overall recovery rate was 77.9%. This finding indicates that our study was not limited to the surgical cohort alone and that a substantial proportion of the overall radial nerve palsy population achieved recovery. Nevertheless, the lower recovery rate in the surgical group and the lower overall recovery rate compared with some published series may be explained by the more complex characteristics of our study population. In the surgical cohort, unfavorable factors such as open fractures, high-energy trauma, new-onset postoperative radial nerve palsy, the need for additional surgery, and more pronounced fracture angulation may have contributed to the lower recovery rate. In addition, because this study was conducted in a teaching and research hospital, surgical procedures were performed under specialist supervision but with the participation of surgeons with different levels of experience, which may have contributed to heterogeneity in the surgical process. However, surgeon experience, primary operator level, and the learning curve were not analyzed as separate variables in this study; therefore, these factors should be considered only as possible contributing factors rather than direct causal explanations. Accordingly, when interpreting the results of this study, it should be kept in mind that conservatively managed cases and surgically treated, more complex cases may have different prognostic profiles.

Regarding the time to recovery, the mean time to recovery of radial nerve function in the surgically treated group was 136.9 days. This corresponds to approximately 4–5 months and is consistent with the recovery times reported in the literature. Bumbasirevic et al. reported initial clinical signs of recovery at a mean of 6 weeks and full recovery at a mean of 17 weeks [18]. Ring et al. reported that, in high-energy injuries, the first signs of recovery appeared at a mean of 7 weeks and full recovery occurred at a mean of 6 months [16]. Because radial nerve recovery may occur over a period ranging from weeks to months, the absence of early recovery signs, particularly in closed fractures, should not always be interpreted as permanent nerve damage. However, open fractures, high-energy injuries, suspected nerve transection, and new-onset postoperative palsy require more careful and active evaluation.

In our study, new-onset postoperative radial nerve palsy was identified in 29 of the 129 surgically treated patients without preoperative radial nerve palsy, corresponding to a rate of 22.5%. Reported rates of postoperative or secondary radial nerve palsy vary in the literature. Schwab et al. reported a secondary radial nerve palsy rate of 6% among 151 patients treated with internal fixation and noted that most of these cases occurred after plate fixation [3]. In the systematic review by Hendrickx et al., the rate of secondary radial nerve palsy in closed humeral shaft fractures was reported as 4% after operative treatment and 0.4% after nonoperative treatment [15]. The rate of new-onset postoperative radial nerve palsy in our study appears higher than the rates reported in the literature. However, this rate should not be interpreted as the rate of permanent or irreversible radial nerve palsy. Most postoperative radial nerve palsies observed in our cohort were clinically consistent with transient neuropraxic injuries rather than complete nerve disruption, as evidenced by spontaneous recovery in 75.9% of cases. Potential mechanisms include intraoperative traction, manipulation around the spiral groove, temporary compression by retractors, postoperative edema, or neurapraxia related to fracture reduction. In fact, radial nerve function recovered clinically in 22 of the 29 patients who developed postoperative radial nerve palsy, corresponding to a recovery rate of 75.9% in this subgroup. The mean time to recovery among these patients was 121.2 days. Accordingly, the rate of persistent postoperative radial nerve palsy among all surgically treated patients without preoperative radial nerve palsy was 5.4%. These findings suggest that a substantial proportion of postoperative radial nerve palsies observed in our study were transient or had the potential for recovery. Therefore, although the rate of postoperative palsy appears high, the rate of persistent postoperative deficit was considerably lower and more comparable with the range reported in the literature.

The decision to perform radial nerve exploration was individualized according to fracture characteristics, the presence of open injury, suspected nerve entrapment, intraoperative findings, progressive neurological deterioration, and surgeon judgment. Routine exploration was not performed in every patient. The necessity and timing of radial nerve exploration remain controversial in the literature. In primary radial nerve palsy associated with closed humeral shaft fractures, many studies support an initial wait-and-see strategy with clinical follow-up. Current evidence generally recommends an initial observation period of approximately 3–4 months for closed fractures with primary radial nerve palsy, provided that serial clinical examinations demonstrate no deterioration and there is no evidence of nerve transection [1,15,18]. In contrast, early exploration is more strongly recommended in the presence of open fractures, vascular injury, penetrating trauma, high-energy injury, suspected nerve entrapment at the fracture site, or suspected nerve transection [1,16,17]. Opinions are more variable regarding new-onset postoperative radial nerve palsy. Schwab et al. reported that in cases where the radial nerve was not visualized during the initial surgery and postoperative palsy developed, early exploration could identify potentially correctable causes [3]. In our study, radial nerve exploration was not significantly associated with nerve recovery. However, this finding should not be interpreted as evidence that exploration is ineffective. The decision to perform exploration was not randomized and may have been preferred in more severe, open, displaced, or high-risk cases. Therefore, the relationship between exploration and outcome may have been influenced by patient selection, injury severity, and surgical indications.

In our study, patients without recovery had longer operative times and higher initial Cobb angles. Longer operative time should not be interpreted as a direct cause of impaired nerve recovery; rather, it may represent an indirect marker of more complex fracture morphology, surgical difficulty, soft tissue injury, and the need for greater manipulation. Similarly, a higher Cobb angle indicates more pronounced angulation and deformity between fracture fragments. This may increase the risk of traction, stretching, contusion, or mechanical irritation of the radial nerve at the fracture site. Hegeman et al. reported that fracture location, fracture pattern, injury energy, and type of surgical fixation may be associated with the development of radial nerve palsy [14]. Lee et al. also reported that radial nerve palsy was more common in younger patients, AO type B fractures, and distal one-third fractures [20]. These findings support the clinical relevance of fracture morphology in both the development of radial nerve palsy and the recovery process.

One of the most important findings of our study regarding functional outcomes was the strong association between recovery of radial nerve function and QuickDASH scores. Patients without nerve recovery had significantly worse QuickDASH scores. In the multivariable analysis, when age, operative time, Cobb angle, and open fracture were included in the model, recovery of radial nerve function was the only significant factor associated with the QuickDASH score. This finding indicates that functional outcome after humeral shaft fracture cannot be explained solely by fracture union, reduction quality, or implant stability; recovery of radial nerve function is also critical for patient-reported upper extremity function. Van Bergen et al. reported generally favorable outcomes at 12 months in patients with radial nerve palsy in terms of DASH score, pain, quality of life, return to activity, and range of motion [19]. In our study, however, the markedly worse functional outcomes in patients without nerve recovery support the clinical relevance of radial nerve recovery as an important outcome parameter.

The need for additional surgery is also important in demonstrating the clinical burden of this patient group. In our study, 49.0% of surgically treated patients required additional surgery, and some patients underwent more than one procedure at different time points. These procedures included nerve grafting, tendon transfer, surgery for nonunion, plate fixation after external fixation, revision open reduction and internal fixation, and additional fixation for non-humeral fractures. Rasulic et al. reported that preserved nerve continuity, low-energy trauma, and surgical treatment within 6 months were associated with better functional outcomes in radial nerve injuries [21]. Temiz et al. also stated that denervation time, the severity of nerve injury, graft length, and surgical technique were important factors affecting recovery in radial nerve injuries [22]. These findings indicate that in complex humeral shaft fractures with radial nerve palsy, not only the initial fracture surgery but also possible reconstructive procedures during follow-up are important for functional outcomes.

This study has several limitations. First, it was designed as a retrospective, single-center study, and the data were obtained from routine clinical records, patient files, operative notes, and radiological archives. Because of the retrospective design, treatment allocation, timing of surgery, indications for radial nerve exploration, and postoperative rehabilitation were determined according to surgeon preference and clinical judgment rather than predefined study protocols. Consequently, selection bias, information bias, and residual confounding cannot be excluded. Furthermore, retrospective data collection relies on the accuracy and completeness of medical records, which may have resulted in incomplete documentation of some clinical variables. Therefore, the associations identified in this study should not be interpreted as causal relationships but rather as clinically relevant observations requiring confirmation in prospective multicenter studies. The surgically treated cohort included patients with heterogeneous injury characteristics, including primary and postoperative radial nerve palsy as well as open and closed fractures. Because of the relatively limited sample size, separate subgroup analyses would have substantially reduced statistical power. Therefore, the present findings should be interpreted within the context of this clinical heterogeneity. Larger prospective studies are warranted to evaluate these subgroups independently. Second, in our center, functional scores such as the QuickDASH are routinely recorded in surgically treated patients, whereas they are not routinely collected in conservatively managed patients. In addition, variables such as operative time, radial nerve exploration, and intraoperative findings are available only for surgically treated patients. Therefore, conservatively managed patients with radial nerve palsy were included in the calculation of overall incidence and nerve recovery rates but were not included in the final functional analyses or in the analyses of surgical variables. This limits direct functional comparison between the surgical and conservative groups. Third, the limited sample size may have reduced the statistical power to detect certain associations, particularly in multivariable analyses. Fourth, EMG findings were missing or obtained at heterogeneous time points in some patients; therefore, the detailed relationship between neurophysiological recovery and clinical or functional recovery could not be fully evaluated. Electromyographic evaluations were not performed according to a standardized protocol but were obtained selectively according to clinical indications at variable follow-up intervals. Consequently, longitudinal electrophysiological recovery could not be systematically analyzed or correlated with functional outcomes. Finally, because radial nerve exploration was performed according to clinical judgment rather than random allocation, patients undergoing exploration likely represented more severe injuries. This selection bias should be considered when interpreting the lack of association between exploration and recovery.

## 5. Conclusions

In conclusion, radial nerve palsy appears to occur more frequently in patients with open humeral shaft fractures than in those with closed fractures. Although recovery rates were high among conservatively managed patients, surgically treated patients demonstrated lower recovery rates, which may reflect the greater complexity and severity of these injuries rather than the effect of treatment itself. Overall, radial nerve function recovered in approximately three-quarters of patients.

Among surgically treated patients, open fractures, longer operative time, and greater initial fracture angulation appeared to be associated with an increased likelihood of persistent radial nerve dysfunction. Furthermore, recovery of radial nerve function was associated with better patient-reported upper extremity function, suggesting that neurological recovery may represent an important determinant of functional outcome.

These findings support the importance of careful neurological assessment, structured follow-up, appropriate rehabilitation, and individualized decision-making regarding radial nerve exploration in patients with humeral shaft fractures. Future prospective, multicenter studies with larger and more homogeneous patient populations are warranted to validate these findings and to better define the optimal management strategy for radial nerve palsy associated with humeral shaft fractures.

## Figures and Tables

**Table 1:** Demographic and clinical characteristics of the patients.

Variable	Value
Age (years), median (IQR)	33 (22.5–50.5)
Sex, *n* (%)	
Female	16 (32.7)
Male	33 (67.3)
Injury energy, *n* (%)	
Low-energy	18 (36.7)
High-energy	31 (63.3)
Fracture type, *n* (%)	
Open fracture	13 (26.5)
Closed fracture	36 (73.5)
Timing of radial nerve injury, *n* (%)	
Post-traumatic	20 (40.8)
Postoperative	29 (59.2)
Treatment modality, *n* (%)	
Plate fixation	47 (95.9)
Intramedullary nailing	2 (4.1)
Fracture pattern, *n* (%)	
Transverse	14 (28.6)
Spiral/oblique	17 (34.7)
Comminuted	18 (36.7)
Cobb angle (°), median (IQR)	24 (16–33.5)
Operative time (min), median (IQR)	100 (80–120)
Nerve exploration, *n* (%)	
Yes	34 (69.4)
No	15 (30.6)
Additional surgery, *n* (%)	
Yes	24 (49.0)
No	25 (51.0)
Recovery of nerve function, *n* (%)	
Yes	35 (71.4)
No	14 (28.6)
Time to recovery (days), mean (min–max)	136.9 (21–500)
QuickDASH score, median (IQR)	37.0 (22.9–55.5)

IQR: Interquartile range, QuickDASH: Quick Disabilities of the Arm, Shoulder and Hand questionnaire.

**Table 2:** Comparison of QuickDASH scores according to clinical characteristics.

Variable	Group	*n*	Mean Rank	*p* Value
Sex	Female	16	26.84	0.529
	Male	33	24.11	
Injury energy	Low-energy	18	22.97	0.449
	High-energy	31	26.18	
Fracture type	Closed	36	25.08	0.946
	Open	13	24.77	
Timing of radial nerve injury	Post-traumatic	20	28.55	0.149
	Postoperative	29	22.55	
Nerve exploration	No	15	22.90	0.494
	Yes	34	25.93	
Additional surgery	No	25	22.38	0.190
	Yes	24	27.73	
Recovery of nerve function	No	14	34.36	0.004 *
	Yes	35	21.26	

Data are presented as mean ranks. Comparisons between groups were performed using the Mann–Whitney U test. QuickDASH: Quick Disabilities of the Arm, Shoulder and Hand questionnaire.* indicates statistical significance (*p* < 0.05).

**Table 3:** Factors associated with recovery of radial nerve function.

Variable	Recovery Achieved (*n* = 35)	No Recovery (*n* = 14)	*p* Value
Sex, *n* (%)			0.501
Female	10 (28.6)	6 (42.9)	
Male	25 (71.4)	8 (57.1)	
Injury energy, *n* (%)			0.202
Low-energy	15 (42.9)	3 (21.4)	
High-energy	20 (57.1)	11 (78.6)	
Fracture type, *n* (%)			0.031 *
Closed fracture	29 (82.9)	7 (50.0)	
Open fracture	6 (17.1)	7 (50.0)	
Timing of radial nerve injury, *n* (%)			0.524
Post-traumatic	13 (37.1)	7 (50.0)	
Postoperative	22 (62.9)	7 (50.0)	
Treatment modality, *n* (%)			1.000
Plate fixation	33 (94.3)	14 (100)	
Intramedullary nailing	2 (5.7)	0 (0)	
Fracture pattern, *n* (%)			0.168
Transverse	11 (31.4)	3 (21.4)	
Spiral/oblique	14 (40.0)	3 (21.4)	
Comminuted	10 (28.6)	8 (57.1)	
Nerve exploration, *n* (%)			0.502
Yes	23 (65.7)	11 (78.6)	
No	12 (34.3)	3 (21.4)	
Additional surgery, *n* (%)			0.217
Yes	15 (42.9)	9 (64.3)	
No	20 (57.1)	5 (35.7)	
Age (years), median (IQR)	30 (22–47)	41.5 (30–65)	0.132
Operative time (min), median (IQR)	90 (75–110)	120 (100–140)	0.033 *
Cobb angle (°), median (IQR)	23 (15–30)	33.5 (21–45)	0.026 *
Distal fracture distance (mm), median (IQR)	90 (73.5–137)	74.5 (48–110)	0.124
Proximal fracture distance (mm), median (IQR)	147 (130–179)	145 (98–180)	0.370
QuickDASH score, median (IQR)	34.0 (22.4–46.5)	72.7 (45.5–79.5)	0.004 *

IQR: Interquartile range, QuickDASH: Quick Disabilities of the Arm, Shoulder and Hand questionnaire. Fisher’s exact test was used for categorical variables, and Mann–Whitney U test was used for continuous variables. * indicates statistical significance (*p* < 0.05).

**Table 4:** Multivariable linear regression analysis of factors associated with the QuickDASH score.

Variable	B (95% CI)	Standardized β	*p* Value
Age	0.303 (−0.027 to 0.633)	0.255	0.071
Operative time (min)	0.021 (−0.181 to 0.222)	0.027	0.838
Cobb angle (°)	−0.024 (−0.551 to 0.503)	−0.014	0.927
Open fracture	−5.060 (−19.817 to 9.697)	−0.100	0.493
Recovery of radial nerve function	−23.027 (−38.314 to −7.740)	−0.467	0.004 *

Dependent variable: QuickDASH score. * indicates statistical significance (*p* < 0.05).

## Data Availability

The data supporting the findings of this study are not publicly available due to privacy and ethical restrictions. The data may be made available from the corresponding author upon reasonable request and with permission from the relevant institutional authorities.

## Abbreviations

CI	Confidence interval
EMG	Electromyography
IQR	Interquartile range
ORIF	Open reduction and internal fixation
QuickDASH	Quick Disabilities of the Arm, Shoulder and Hand
RNP	Radial nerve palsy
SD	Standard deviation
